# Editorial: Metastable Dynamics of Neural Ensembles

**DOI:** 10.3389/fnsys.2017.00099

**Published:** 2018-01-26

**Authors:** Emili Balaguer-Ballester, Ruben Moreno-Bote, Gustavo Deco, Daniel Durstewitz

**Affiliations:** ^1^Department of Computing and Informatics, Faculty of Science and Technology, Bournemouth University, Poole, United Kingdom; ^2^Bernstein Center for Computational Neuroscience Heidelberg-Mannheim, Mannheim, Germany; ^3^Center for Brain and Cognition and Department of Information and Communications Technologies, Pompeu Fabra University, Research Unit, Parc Sanitari Sant Joan de Déu, Barcelona, Spain; ^4^Center for Brain and Cognition, Computational Neuroscience Group, Department of Information and Communication Technologies, Universitat Pompeu Fabra, Barcelona, Spain; ^5^Institució Catalana de la Recerca i Estudis Avançats (ICREA), Barcelona, Spain; ^6^Department of Neuropsychology, Max Planck Institute for Human Cognitive and Brain Sciences, Leipzig, Germany; ^7^School of Psychological Sciences, Monash University, Melbourne, VIC, Australia; ^8^Department of Theoretical Neuroscience, Bernstein Center for Computational Neuroscience Heidelberg-Mannheim, Central Institute of Mental Health, Medical Faculty Mannheim, Heidelberg University, Mannheim, Germany

**Keywords:** metastability, trial-to-trial variability, transient dynamics, attractor dynamics, neural noise

A classical view of neural computation is that it can be characterized in terms of convergence to fixed-point-type attractor states (representing for instance memory patterns in Hopfield, 1982) or limit-cycle-like sequential transitions among states (mapping e.g., motor or syntactical sequences in Elman, 1990). After over three decades, is this still a valid model of how brain dynamics implements cognition? The idea that neuro-computational dynamics is mainly deterministically driven by convergence to emergent stable states in a synaptic/network noisy background has been lively debated, and recently challenged both empirically and by computational work. This question touches on the very basics of our understanding of neural computation; and hence it is one of the most exciting topics currently in systems and computational neuroscience.

This e-book comprises a comprehensive collection of recent theoretical and experimental contributions addressing the question of stable versus transient neural population dynamics, and its implications for the observed variability in neural activity, from diverse, complementary angles.

## Metastability in models

A connecting theme for the multiple contemporary views on metastability in the brain was proposed first by Tognoli and Kelso. In their foundational approach, the authors discuss classical and recent views on how information transfer between brain regions could be accomplished through synchronization and collective neural responses. They frame these ideas in terms of the *coordination dynamics* concept, potentially a key aspect for understanding metastability in neuronal populations.

Metastability and its possible functional role both within and outside of behavioral task contexts is further addressed in four specific modeling approaches situated at different spatial scales, ranging from macro/mesoscopic levels (Schwappach et al.; Stratton and Wiles; Aguilera et al.) to a biophysically detailed level of neuronal systems description (Mazzucato et al.).

The balance between global segregation and integration at a macroscopic scale is theoretically analyzed by Stratton and Wiles. They propose a computational model focused on how the thalamo-cortical loop may underlie long-range segregation between brain regions, producing metastable responses observed at large spatial scales. Metastability at macroscopic levels could also stem from sensimotor interactions, as suggested by Aguilera et al. These authors designed a new theoretical framework and implemented it in an agent-based model which interacts with the environment. According to this model, metastability arises from the dynamics of sensori-motor feedback interactions, beyond what would be expected from considering brain activity just in isolation.

At mesoscopic scales, neural population models have been constructed that produce metastability through attracting chains of heteroclinic orbits, generating transient dynamics through a sequence of saddle points along which one or several axes are stable (the stable subspace). Following up on this theory, Schwappach et al. demonstrate, using a novel neural field model, how such heteroclinic subspaces can account for part of the observed trial-to-trial variability at the mesoscopic level. Hence, this variability may partly stem from sources other than neuronal or synaptic noise.

At microscopic (biophysical) scales, using a clustered spiking model (in which connectivity patterns are heterogeneous) which exhibits metastable states, Mazzucato et al. show that variation in neuronal ensemble activity may be confined to small subspaces of the whole state space spanned by all the individual units' firing-rates. Moreover, the dimensionality of these subspaces is smaller during stimulus-evoked activity than in the absence of a task. This is in line with empirical studies which report the reduction of neuronal variability upon stimulus presentation (Churchland et al., 2010).

## Empirical studies

Metastability was also addressed in four studies which provide novel analytical tools and empirical evidence. Tošić et al. proposed a new data analysis technique to identify metastability empirically, which was used to infer metastable states in local field potentials evoked by visual stimuli in anesthetised ferrets.

Interestingly, *visual scan paths* (Wilkinson and Metta) reveal complex dynamics which possibly reflects underlying metastable neural activity. In Wilkinson and Metta, the authors proposed a theoretical framework, termed the *singularity hypothesis*, which relies on transient spiral waves which govern persistent neural activity states underlying oculomotor postural control. In general, motor control strategies may be represented in neuronal activity patterns as a complex, distributed spatiotemporal code, which may not be revealed by looking just at neuronal firing rates within recorded ensembles. This is shown in Mao et al. who study behaving rats performing a directional choice task, using a nonlinear decoder to demonstrate how spatiotemporal activity patterns in motor areas increasingly discriminate the animal's choices as learning progresses.

Finally, spatiotemporal patterns generated by synchronized spontaneous activity in the idle brain are analyzed at different spatial scales in two studies (Liu et al.; Yada et al.) which further illustrate the rich diversity in methodological approaches to the empirical identification of metastable and transient dynamics. Specifically, Yada et al. propose that repeating spatiotemporal patterns emerging during synchronized bursts of activity *in vitro* may have their origin in specific sub-populations that become sequentially active in a reproducible temporal order. This result suggests an orchestrated activation of such ensembles that depends on the global state of the network, consistent with spontaneous transitions between metastable states.

At a macroscopic level, Liu et al. develop a new method for identifying co-activity patterns of fMRI responses which is robust to non-stationarity. In this study, a novel type of cluster analysis suggests a richer repertoire of co-activation states beyond the resting-state networks identified previously, and hence perhaps a more specialized functional organization.

In summary, this book provides a comprehensive collection of current modeling and data analysis approaches related to the metastable behavior of cortical ensembles. These studies showcase recent efforts for designing a fundamental framework that encompasses the multiple facets of metastability in neural responses, beyond the original use of the concept in the context of statistical mechanics. To conclude, the last chapter of the book reflects on this plethora of approaches and connects them with the question of the functional role of experimentally observed trial-to-trial variability (Balaguer-Ballester), one of the most intriguing topics currently in systems neuroscience (Moreno-Bote, 2014).

## Author contributions

All authors listed have made a substantial, direct and intellectual contribution to the work, and approved it for publication.

### Conflict of interest statement

The authors declare that the research was conducted in the absence of any commercial or financial relationships that could be construed as a potential conflict of interest.
